# Stenting of high-tortuous ducts in duct-dependent pulmonary circulation: essential points to consider before deciding on stenting

**DOI:** 10.3389/fcvm.2023.1275545

**Published:** 2023-11-20

**Authors:** Nathalie Mini

**Affiliations:** Cardiac Catheterization Laboratory, German Paediatric Heart Centre, University Hospital of Bonn, Bonn, Germany

**Keywords:** duct-dependent pulmonary circulation, duct-stenting, tortuous duct, pulmonary branch stenosis, stent failure, ductal curvature index

## Abstract

Despite the advancements in the technique of duct stenting (DS) in patients with duct-dependent pulmonary circulation (DDPC) and the valuable role of DS in preventing the risk of surgical creation of shunts and early repair, not all ducts are amenable to being stented, and not all interventions with DS are safe and can achieve positive outcomes. Very few studies focusing on tortuous ducts have been conducted until now. Their results showed that stenting of highly tortuous ducts has the same risk as surgical options. This type of stenting has greater possibility of complications, early in-stent thrombosis, and stent failure than do other duct types. In such cases, the surgical options could be superior to DS and have better outcomes. This report aims to review the very scarce available data about stenting of high-tortuous ducts and criticisms of performing DS in ducts associated with pulmonary stenosis and to highlight the essential points that must be considered before deciding on intervention.

## 1. Introduction

The feasibility of duct stenting (DS) in patients with duct-dependent pulmonary circulation (DDPC) and its efficacy as a temporary measure prior to planned surgery has led DS to be the first option in many care centres worldwide (1–3). However, not all ducts are amenable to being stented, and not all interventions with DS are safe and can achieve a positive outcome (4–6). For more than 25 years, many studies have shown that DS could be an effective alternative to the modified Blalock-Taussig shunt (MBTS). Still, very few studies focused on the causes of stenting failures in patients who underwent DS and had negative outcomes (7–9). In 2019 (5), the ducts were classified based on their morphology. Subsequently, in 2021, the first trial to find a threshold cut-off was conducted (6), suggesting that the ducts with ductal curvature index (DCI) equal to or greater than 0.45 should be considered high-risk ducts for stenting.

This report aims to review the types of ducts in patients with DDPC based on the associated congenital heart failure and to highlight the points to consider regarding the stenting of high-tortuous ducts before deciding to intervene.

### 1.1. How does the duct differ morphologically depending on the heart defects?

#### 1.1.1. Pulmonary atresia with the intact ventricular septum

**Figure 1:**
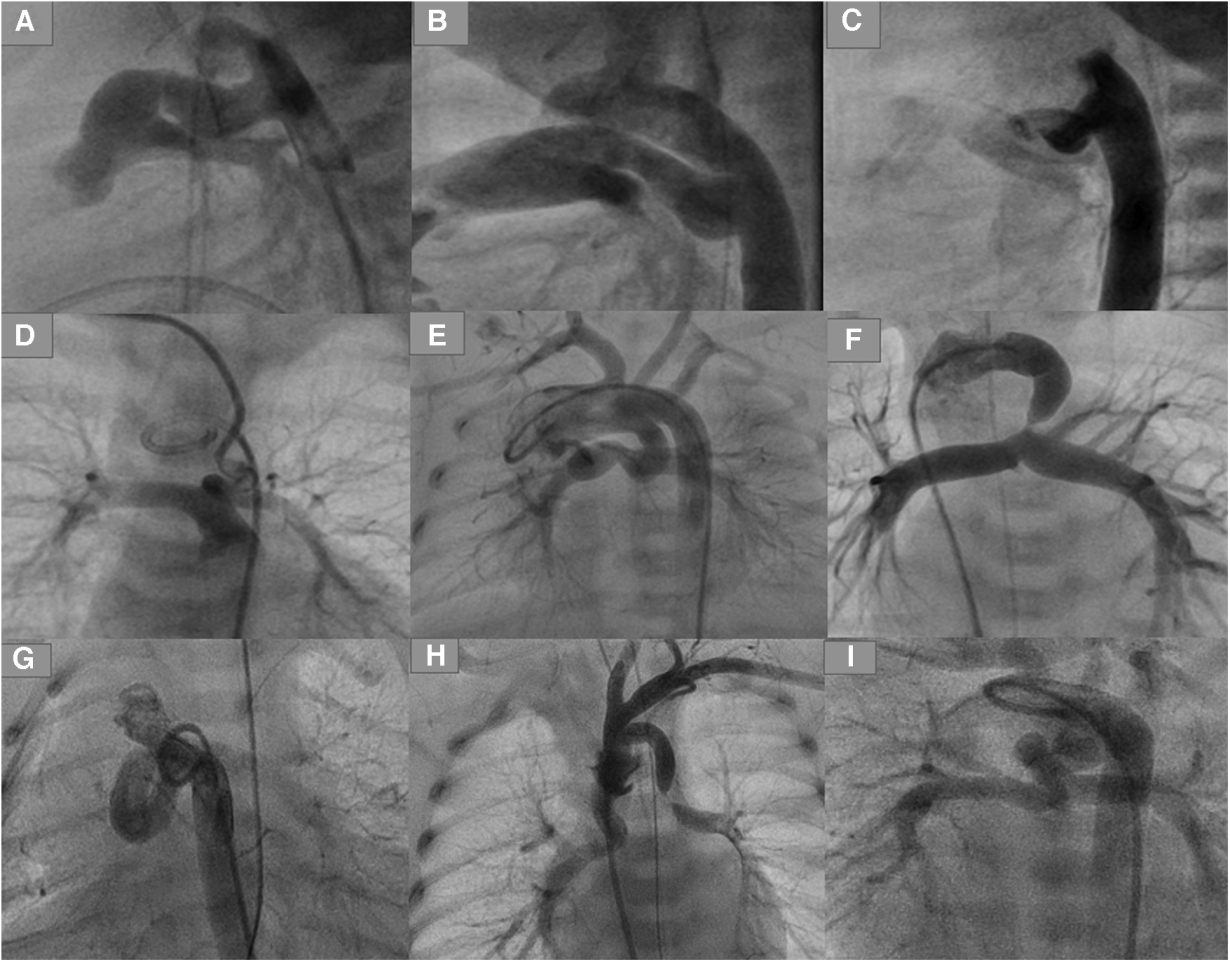
Types of ducts in congenital heart disease with duct-dependent pulmonary circulation. (**A,B**) Typical ducts originate from DAO in PA-IVS. (**C**) Tortuous duct originates from DAO with multiple complex curves. (**D,E**) Ducts originate from aortic arch with cervical course. (**F**) Duct originates from left subclavian artery. (**G**) Tortuous duct originates from DAO proximal to aortic arch. (**H**) Double ducts, the first originates from left subclavian artery and the second from the aortic arch. (**I**) Tortuous duct originates from aortic arch with multiple complex curves.

The duct associated with pulmonary atresia (PA) or critical pulmonary stenosis (PS) with an intact ventricular septum (IVS) is usually straight with no or only a mild curvature. In sporadic cases, one or more curves have been seen. The duct originates from the descending aorta and inserts into the pulmonary trunk, usually without pulmonary branch stenosis (Figure 1). These ducts can be crossed anterogradely or retrogradely when the PA is opened or when the stenosis is crossed.

#### 1.1.2. Pulmonary atresia with ventricular septum defect

The duct associated with PA or PS and ventricular septum defect (VSD) varies in morphology depending on the associated heart defects and the origin of the duct. There is a significant relationship between the severity of tortuosity and related heart failure (4). The duct in PA-VSD can originate from the descending aorta, the underside of the aortic arch (opposite the subclavian or the carotid artery), or the subclavian artery/truncus brachiocephalic.

A duct originating from the subclavian artery with insertion into the left pulmonary artery (LPA) will be seen more frequently in tetralogy of Fallot (TOF), with different double outlet right ventricle (DORV) variants, including single ventricle (e.g., right isomerism). Such ducts are usually long, mild to moderate tortuous, and require more than one stent. In some cases, coarctation or sub-coarctation of pulmonary arteries has been observed. In sporadic cases, a second duct originating from the aortic arch has been observed, with potential insertion into the other pulmonary artery (Figure 1).

Ducts originating from the underside of the aortic arch tend to be torturous and have one, two, or multiple complex curves with pulmonary branch stenosis or coarctation. These ducts can be seen frequently with TOF and DORV with Dextro-Transposition of the Great Arteries (dTGA). These types of ducts, when opposite the carotid arteries, will often have a vertical course and insertion into the LPA, right pulmonary artery (RPA), or pulmonary trunk.

The tortuosity of such ducts varies depending on the length of the duct and the subsequent required curve between the origin and the duct insertion. The longer the duct, the greater the ductal curvature and tortuosity. Axillary or carotid access is to be required for crossing such ducts. Uncommonly, the duct inserts equally into both pulmonary arteries in case of the absence of the pulmonary trunk; two stents can be required for sufficient perfusion of both arteries in such cases (Figure 1).

Ducts originating from the descending aorta are less common in PA-VSD than in PA-IVS and have an S-shape configuration with one or multiple complex curves. Unilateral or bilateral pulmonary branch stenosis caused by ductal tissue has often been seen. This type of duct is associated more frequently with double inlet left ventricle (DILV) with transposition of the great vessels (TGA), PA with tricuspid atresia, and DORV with malposition of the great arteries (MAG) or TGA (Figure 1).

The origin of such ducts can be more proximal to the aortic arch in cases of PA-VSD than in cases of ducts seen in PA-IVS, which requires crossing the duct via the subclavian or carotid artery due to the sharp angulation caused between the aorta and the origin of the duct.

### 1.2. How are the ducts classified morphologically depending on tortuosity?

Qureshi et al. classified the ducts into three types depending on the tortuosity (5). Type 1 includes straight ducts, which have no or a mild curve. Type 2 includes ducts with one curve. Type 3 includes multiple complex curves. Their results showed that the pulmonary branch stenosis and the need for reintervention were higher in ducts with higher tortuosity indexes.

### 1.3. What is the ductal curvature index?

**Figure 2:**
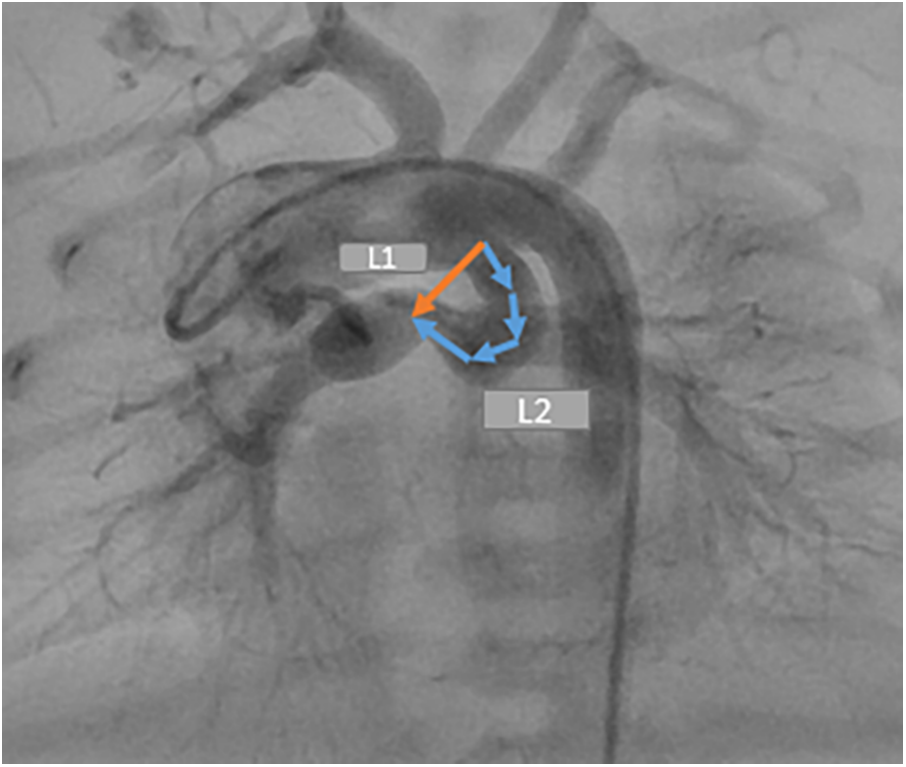
Demonstration of two distances required for measuring the ductal curvature index. DCI = L2–L1/L2.

The DCI is a quantitative measurement of ductal tortuosity first reported by Qureshi et al. in 2019. It determines the severity of the ductal tortuosity. Two distances are required to measure DCI (L2 and L1). L2 is the entire length between the ductal origin and the ductal insertion onto the pulmonary arteriy. L1 is the straight, short distance between the two points (Figure 2).

### 1.4. What is the cut-off threshold of the ductal tortuosity?

In 2021, the first study proposed a quantitative cut-off threshold for ductal tortuosity using DCI in 71 patients with true DDPC (6). Mini et al. (6) suggested that a clinical cut-off point based on the severity of ductal curvature (DCI ≥ 0.45) may identify high-risk patients for stenting in whom DS may be insufficient as an intermediate measure prior to a planned surgery or intervention. The results showed that DCI ≥ 0.45 could be a statistical cut-off point that could predict the outcome of DS depending on the DCI. Such information could help to identify and distinguish the high-risk ducts for stenting, in which the incidence of early, midterm, and late stent failure could be very high. Only 20% of ducts with DCI ≥ 0.45 achieved the primary outcome compared with 93% of those with DCI < 0.45. The results showed a significant relationship between DCI and early complications, late complications, morbidity, and mortality.

### 1.5. What are the early and late complications that could result from stenting of tortuous ducts?

Ductal spasms while crossing the duct and acute in-stent thrombosis could be life-threating situations. Dislocation of the stent, duct dissection, or dissection of the accessed artery (carotid or axillary arteries) have been documented (6). Early in-stent stenosis and thrombosis have been seen frequently in the weeks after stenting due to deformation of a stent, stent overexpansion, stent underexpansion, and use of long stents in tortuous vessels (10, 11). The deformation of the stent in the tortuous duct makes the reintervention in the stent complex and highly risky. Compared with ducts with DCI < 0.45, ducts with DCI ≥ 0.45 showed an increase in major complications from 1.7% to 26%, and the need for stent-failure-related surgery increased from 1.7% to 60% (6).

### 1.6. What do we know about the impact of duct-stent failure on the outcome of modified blalock-taussig shunts?

From the literature, we know little about the impact of duct-stent failure on the outcome of MBTSs. A study published in 2022 (12) compared the MBTS and DS in patients with highly tortuous ducts (DCI ≥ 0.45). The results showed that hospital death after creating MBTS increased from 23.5% in patients who received a shunt as a primary option to 38% in patients who underwent DS preoperatively. The impaired outcome rose from 35% to 50% in the same cohort. A shunt was needed due to an early in-stent thrombosis, late-stent thrombosis, or a complex in-stent reintervention. The rest required an MBTS due to insufficient pulmonary perfusion without stent thrombosis and underdevelopment of one or both pulmonary arteries. The desaturation and hemodynamical instability related to a stent failure could lead to fundamental changes in pulmonary vascular resistance, pulmonary development, compliance, and the perfusion of the organs due to low cardiac output and metabolic changes. Such preoperative conditions will be unsuitable for optimal intraoperative and postoperative surgery results, undermining the surgical outcome by increasing morbidity and mortality (5, 6, 12).

### 1.7. What criticisms surround stenting a tortuous duct with significant pulmonary stenosis, and what are the points to consider in such cases?

Stenting a tortuous duct associated with significant pulmonary branch stenosis is controversial and should be carefully evaluated before intervening. The cardiologist must consider several points for better decision-making in such ducts.

**Figure 3:**
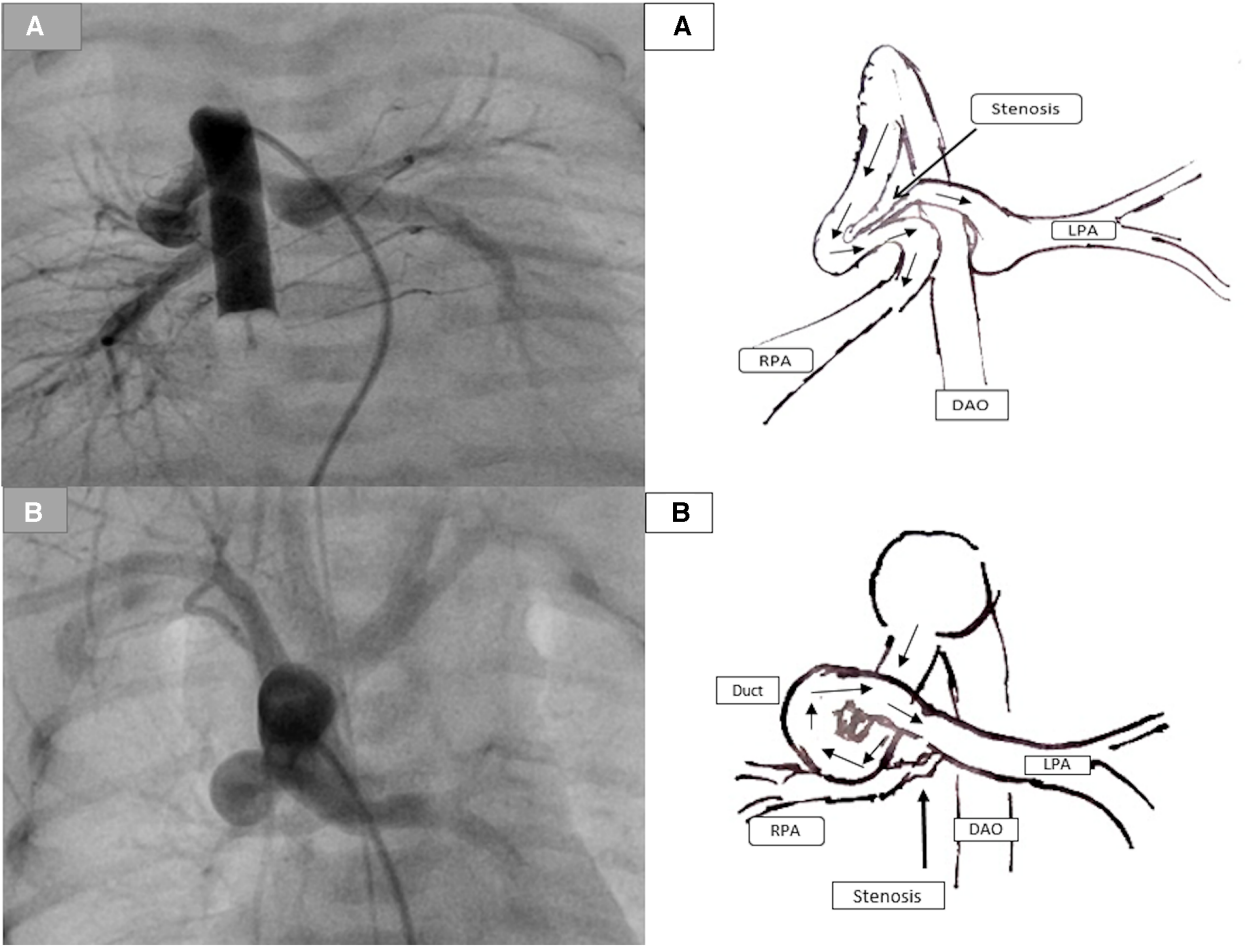
Demonstration of the duct-associated pulmonary stenosis. (**A**) represents a tortuous duct with two curves with significant pulmonary stenosis (PS) caused by the ductal tissue. (**B**) represents a tortuous duct with complex curves and significant RPA stenosis.

First, stenting the tortuous duct with significant pulmonary branch stenosis, usually the LPA, is challenging and can be complicated. The complex ductal morphology increases the instability of the wire during the attempts to cross the stenotic side of the LPA and the loss of the wire position. Repeating attempts to cross the stenotic pulmonary side increases the risk of acute spasms in the duct or at the entrance of the LPA and acute thrombosis, which can be life-threatening (Figure 3).

Second, duct-associated pulmonary branch stenosis is caused by the ductal tissue at the ductal insertion into the LPA. Stenting of the pulmonary stenosis requires choosing a longer stent and inserting the stent more distally into the pulmonary branch. This could accelerate the stenosis of the LPA, which impacts the outcome of the planned surgery, especially in single-ventricle morphology intended for Fontan circulation.

**Figure 4:**
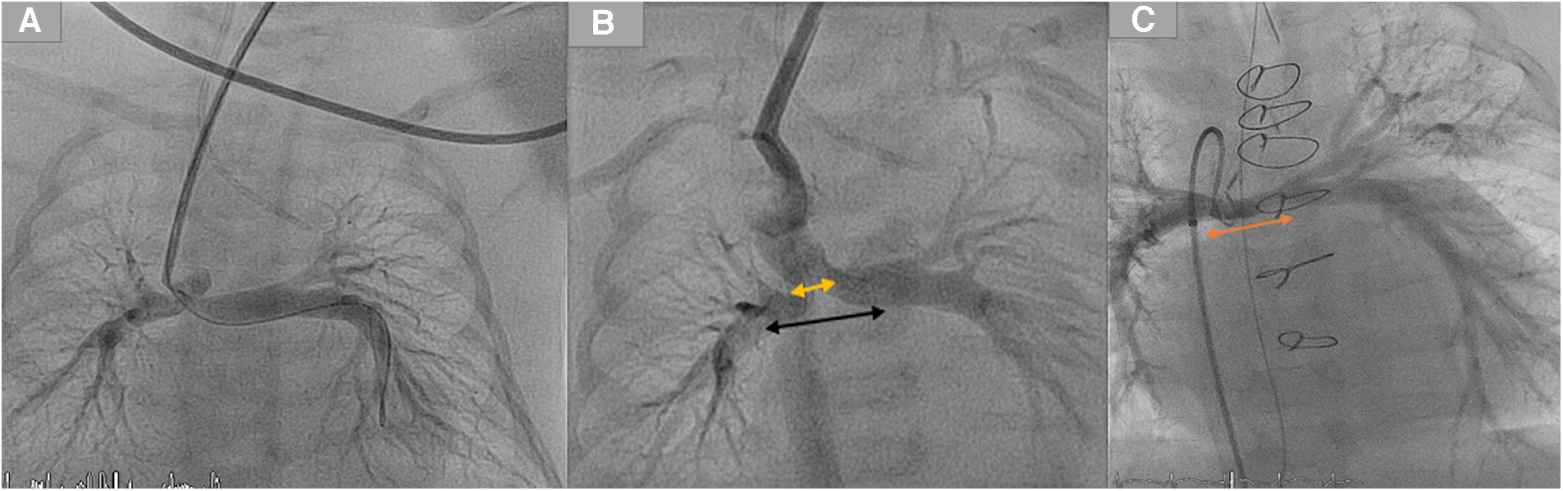
Demonstration of two stents in a duct with mild bilateral pulmonary stenosis. (**A**) Duct originates from the aortic arch and inserts into the pulmonary arteries without the pulmonary trunk. (**B**) Two duct stents were implanted and inserted into both arteries. The orange arrow represents the distance required to reconstruct the pulmonary arteries. In contrast, the black arrow represents the pulmonary segments that were surgically resected by removing the stents after the ductal stent. (**C**) The orange arrow for the same patient represents the underdeveloped central pulmonary arteries after the resection of stents and the pulmonary reconstruction.

Third, in the case of stenting such ducts and the related LPA stenosis, and during the surgical resection of the duct stent and its segment in LPA, the surgeon will have to remove the stent as well as an essential part of the LPA, which can extend to the hilus in a case of a long stent. Subsequently, the required patch will involve, in many cases, the central LPA until the hilus, which will impact the growth of the LPA and the outcome of the Fontan operation in a single ventricle (Figure 4) (7). In sporadic cases of the absence of a pulmonary trunk associated with a vertical duct and bilateral PS, two stents in the duct to both pulmonary arteries will be required. The surgical removal of the stents requires resecting and reconstructing an essential central segment of both pulmonary arteries, undermining their growth (Figure 4).

Several studies (5, 6, 12) showed that the discrepancy between the pulmonary arteries and the requirement of pulmonary reconstruction in tortuous ducts that received stents was equal to those documented in MBTS. That means DS in tortuous ducts associated with pulmonary branch stenosis loses one advantage in improving the pulmonary branches' symmetrical growth.

### 1.8. What about the question “are there contraindications for ductal stenting?”

Because there is no consensus among experts to answer this controversial question because of the common confusion between and the incorrect separation of the concepts of successful stenting of high-tortuous ducts and the long-term outcome of the stenting, this manuscript proposes to replace the above question with the question “When should we critically discuss and balance the pros and cons of DS and the surgical options for a better long-term outcome?”. The general definition of the successful stenting of high-tortuous ducts is to achieve DS with one or more stents without intervention-related complications and sufficient perfusion of both pulmonary arteries at the end of the intervention. This definition is commonly separated from the intervention's follow-up and ductal-stent outcome when speaking about highly tortuous ducts even though not all successfully stented tortuous ducts can provide sufficient perfusion and symmetrical development of both pulmonary arteries in the early, midterm, and late follow-ups. Furthermore, not all successfully stented ducts can serve as intermediate measures for patients prior to their planned surgeries without the need for additional, unplanned surgeries or even for a stent in the right ventricular outflow tract in the case of PA-VSD or TOF. In addition, not all stented ducts can be safely reintervened when acute restenosis or in-stent thrombosis occurs due to a significant stent deformation. Moreover, not all stented ducts can be removed by surgeons without resection and reconstruction of significant parts of the involved pulmonary artery due to a suboptimal stent position.

Based on the above, it is reasonable to revise the previous definition of a successful high-tortuous duct stenting to one that successfully serves as an intermediate measure for a patient prior to a planned surgery with satisfactory development of both pulmonary arteries and without the need for unplanned surgical or interventional options to improve the pulmonary circulation or the need for a significant stent-related pulmonary segment resection and reconstruction.

### 1.9. Why is it recommended to discuss with the surgeon before stenting a high-tortuous duct?

For all the aforementioned reasons regarding high-tortuous ducts, it is essential to discuss with the surgeon the pros and cons of DS compared with the alternative surgical options to make a better decision for better long-term results in this cohort.

Such decisions are not only about a successful stenting in a highly challenging case but also about a successful bridge for the patient until the next surgery with the best possible conditions regarding the growth of pulmonary arteries, the stability of saturation, and avoidance of undesired stress caused by stent failure.

### 1.10. Which patients with duct-dependent pulmonary circulation might be more suitable for surgical options?

**Figure 5:**
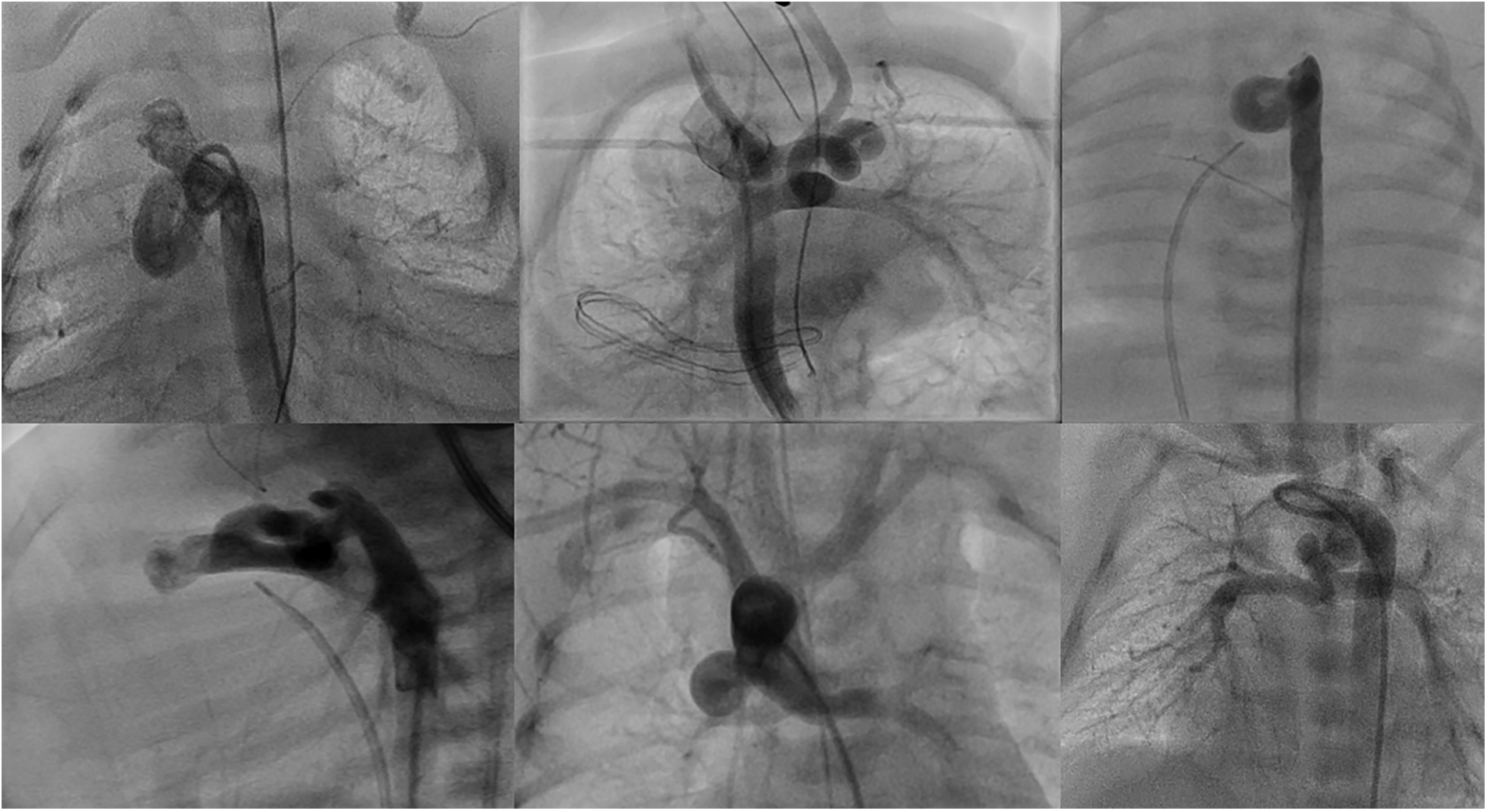
Demonstration of different types of tortuous ducts in patients with duct dependent pulmonary circulation which would be candidates for surgical options.

The following patients who weigh >2.5 kg (in some centres, >3 kg) and who have no contraindications for surgery could benefit from surgical palliation or surgical repair more than intervention:

Patients with single ventricle and tortuous ducts (multiple complex curves with loop or DCI > 0.45) usually associated with pulmonary branch stenosis or pulmonary coarctation, in whom the stent is intended to be at the stenotic side (Figures 3, 5).

In these patients, there is a high likelihood of stent-related complications as well as a suboptimal position of the deformed stent with a high probability of the stent's slipping into the distal part of the pulmonary branch (sometimes up to the hilus). This leads to a subsequent surgical challenge to remove the stent and reconstruct an essential segment of the involved pulmonary branch, which could impair the pulmonary growth. An elective aortic pulmonary shunt could benefit such patients (12).

Patients with a single ventricle and an absent pulmonary trunk with insertion into both pulmonary arteries who will need two stents at both pulmonary branches (Figure 4).

These patients could benefit from surgical options to avoid a large surgical pulmonary anastomosis between the two reconstructed branches after stent resection, which could impair the pulmonary development.

Patients with two-ventricle morphology with high-tortuous ducts (usually associated with pulmonary stenosis or coarctation) (Figure 5)

In these patients, early repair or partial repair could be considered as an alternative to stenting (PA, VSD, and TOF).

### 1.11. What should be investigated further regarding the stenting of tortuous ducts?

We have numerous studies about the excellent outcome of DS as an alternative to MBTS and early repair. However, more studies could focus on patients who had duct-stent failure and needed unplanned surgical options and thus highlight the morbidity, mortality, and outcomes compared with patients who received surgical options in the same cohort. Such focused studies could help identify the stent-failure-related reasons and the durability of stent-patency in tortuous ducts to find better alternatives and adapt suitable stents for such ducts.

## 2. Conclusion

Stenting high-tortuous ducts in patients with DDPC is challenging and controversial due to the unclear data about the outcomes. Despite the brilliant results of DS compared to surgical options in DDPC, not all DS procedures have positive outcome, and not all surgical palliations have impaired outcomes.

Several points should be considered before deciding on the stenting of such ducts:

1. This high-risk intervention is associated with increased occurrence of vascular-access damage, loss of the wire and subsequently the stent, stent malposition, stent deformation, and duct spasms that could be life-threatening conditions.
2. The increased incidence of stent failure with the need for unplanned surgical or other interventions with subsequent undesired stress and hypoxia could impair the outcome of the surgery.
3. The high incidence of the need for reconstruction of significant pulmonary segments due to a slipped stent distal in the pulmonary branch could impair the growth of the pulmonary arteries, especially in patients intended for Fontan circulation.

The surgical interventions for such high-risk patients could be superior in some cases. Thus, such options should be considered and discussed with the surgeons for better decision-making and to achieve better outcomes. Finally, more studies are needed to define a precise cut-off threshold of the DCI to help identify patients at higher risk for stenting.

## References

[1] Glatz AC Petit CJ Goldstein BH Kelleman MS McCracken CE McDonnell A Comparison between patent ductus arteriosus stent and modified blalock-taussig shunt as palliation for infants with ductal-dependent pulmonary blood flow: insights from the congenital catheterization research collaborative. Circulation. (2018) 137(6):589–601. 10.1161/CIRCULATIONAHA.117.029987 29042354

[2] Schranz D Michel-Behnke I Heyer R Vogel M Bauer J Valeske K Stent implantation of the arterial duct in newborns with a truly duct-dependent pulmonary circulation: a single-center experience with emphasis on aspects of the interventional technique. J Interv Cardiol. (2010) 23(6):581–8. 10.1111/j.1540-8183.2010.00576.x 20642476

[3] Schneider M Zartner P Sidiropoulos A Konertz W Hausdorf G. Stent implantation of the arterial duct in newborns with duct-dependent circulation. Eur Heart J. (1998) 19(9):1401–9. 10.1053/euhj.1998.0977 9792267

[4] Alwi M. Stenting the ductus arteriosus: case selection, technique and possible complications. Ann Pediatr Cardiol. (2008) 1(1):38–45. 10.4103/0974-2069.41054 20300236 PMC2840730

[5] Qureshi AM Goldstein BH Glatz AC Agrawal H Aggarwal V Ligon RA Classification scheme for ductal morphology in cyanotic patients with ductal dependent pulmonary blood flow and association with outcomes of patent ductus arteriosus stenting. Catheter Cardiovasc Interv. (2019) 93(5):933–43. 10.1002/ccd.28125 30790426

[6] Mini N Schneider MBE Zartner PA. Use of the ductal curvature index to assess the risk of ductal stenting in patients with duct-dependent pulmonary circulation. Transl Pediatr. (2021) 10(5):1307–16. 10.21037/tp-21-17 34189088 PMC8193002

[7] Alwi M Mood MC. Stenting of lesions in patent ductus arteriosus with duct-dependent pulmonary blood flow: focus on case selection, techniques and outcome. Interv Cardiol Clin. (2013) 2(1):93–113. 10.1016/j.iccl.2012.09.011 28581990

[8] Santoro G Gaio G Palladino MT Castaldi B Iacono C Esposito R Arterial duct stenting: do we still need surgical shunt in congenital heart malformations with duct-dependent pulmonary circulation? J Cardiovasc Med. (2010) 11(11):852–7. 10.2459/JCM.0b013e32833a070d 20442671

[9] Santoro G Capozzi G Caianiello G Palladino MT Marrone C Farina G Pulmonary artery growth after palliation of congenital heart disease with duct-dependent pulmonary circulation: arterial duct stenting versus surgical shunt. J Am Coll Cardiol. (2009) 54(23):2180–6. 10.1016/j.jacc.2009.07.043 19942090

[10] Buccheri D Piraino D Andolina G Cortese B. Understanding and managing in-stent restenosis: a review of clinical data, from pathogenesis to treatment. J Thorac Dis. (2016) 8(10):E1150–62. 10.21037/jtd.2016.10.93 27867580 PMC5107494

[11] Alraies MC Darmoch F Tummala R Waksman R. Diagnosis and management challenges of in-stent restenosis in coronary arteries. World J Cardiol. (2017) 9(8):640–51. 10.4330/wjc.v9.i8.640 28932353 PMC5583537

[12] Mini N Schneider MBE Asfour B Mikus M Zartner PA. Duct stenting vs. Modified blalock-taussig shunt: new insights learned from high-risk patients with duct-dependent pulmonary circulation. Front Cardiovasc Med. (2022) 9:933959. 10.3389/fcvm.2022.933959 35811693 PMC9261874

